# Management of Grade III Femoral Notching During Robotic-Assisted Total Knee Arthroplasty Using a Proximal Tibial Autograft and Countersunk Screw Fixation: A Case Report

**DOI:** 10.7759/cureus.114978

**Published:** 2026-08-22

**Authors:** Ajay Shukla, Omprakash Meena, Amit Kumar, Manjesh Reddy S. V., Deepak Kumar

**Affiliations:** 1 Department of Orthopaedic Surgery, Atal Bihari Vajpayee Institute of Medical Sciences and Dr. Ram Manohar Lohia Hospital, New Delhi, IND

**Keywords:** autograft, femoral notching, proximal tibial cut, robotic, total knee arthroplasty

## Abstract

Femoral notching is an iatrogenic violation of the anterior cortex of the distal femur that occurs during femoral component preparation in total knee arthroplasty (TKA). This cortical breach has been implicated as a risk factor for a future periprosthetic supracondylar fracture, particularly when the defect is deep and compromises structural integrity. Robotic systems may reduce the incidence but do not eliminate this error. We report a case of intraoperative management of a grade III femoral notching during robotic-assisted primary TKA in a 56-year-old female with Kellgren-Lawrence grade IV bilateral knee osteoarthritis and severe varus deformity, with a baseline Knee Society Score (KSS) knee score of 31 and functional score of 20. The defect was reconstructed using an autograft harvested from the proximal tibial cut, which was fashioned to fit the defect and secured with two 3.5 mm countersunk cortical screws. Following reconstruction, standard cruciate-retaining primary femoral and tibial components were implanted. The patient followed a strict non-weight-bearing regimen for four weeks, followed by protected weight-bearing for a further four weeks. At 12 months postoperatively, the patient had a range of motion of 0°-0°-120°(extension-neutral-flexion) with a KSS knee score of 99 and a functional score of 90. The radiographs showed cortical and trabecular continuity at the anterior cortex, indicating autograft incorporation and remodeling. Even though robotic and navigation systems promise precision, they are never without errors, and the need for human skill and expertise should never be overlooked. This report describes the feasibility of reconstruction of a grade III femoral notch using a structural autograft from the proximal tibial resection, avoiding conversion to a stemmed femoral component, use of allograft, or metal augments, with short-term clinical and radiographic follow-up.

## Introduction

Femoral notching represents an unintended cortical breach of the distal femur during preparation for the femoral component in total knee arthroplasty (TKA). Biomechanical studies have demonstrated that notching reduces the torsional and bending strength of the distal femur, predisposing to periprosthetic fracture [1-4]. A recent meta-analysis demonstrated an increased risk, particularly when the notch depth is at least 3 mm [2,5].

Navigation and robotic systems have been developed to improve bone cut precision and component positioning, but they are not without risks depending on planning techniques, referencing, or execution-related errors [3]. The Tayside classification grades femoral notching into four types: Grade I: violation of the outer cortex; Grade II: violation of both the inner and outer tables; Grade III: extension up to 25% of the medullary canal; Grade IV: extension up to 50% of the medullary canal [1]. Currently, there is a lack of clear consensus regarding the management of significant femoral notching. When substantial notching occurs, management options include accepting the defect with strict postoperative weight-bearing restrictions or utilizing a stemmed femoral component.

We describe a novel technique for intraoperative reconstruction of a Tayside Grade III femoral notching during robotic-assisted TKA using structural autograft from the proximal tibial resection and countersunk cortical screw fixation, with 12-month clinical and radiographic follow-up.

This article was previously presented as a poster at the Delhi Orthopaedic Association Annual Meeting on December 27, 2025.

## Case presentation

**Figure 1:**
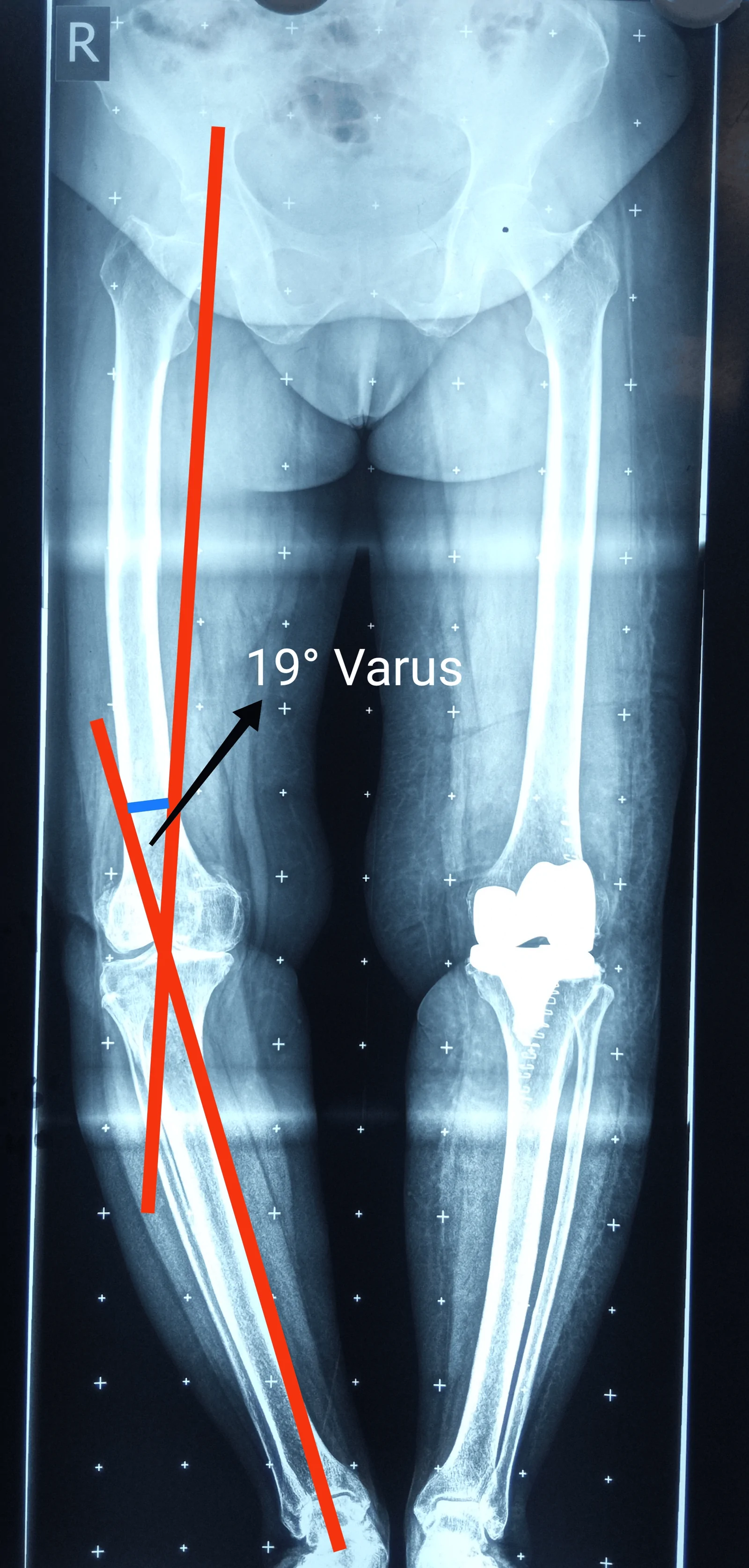
Standing scannogram showing severe osteoarthritis and a 19° varus deformity of the right knee.

A 56-year-old female presented to the orthopaedic outpatient department with severe chronic right knee pain and functional limitation. She had no systemic comorbidities, and her BMI was 22.5 kg/m^2^. Examination revealed severe varus deformity of the right knee and range of motion of 0°-0°-80°(extension-neutral-flexion). Radiographs showed reduced joint space and osteophyte formation, suggestive of Kellgren-Lawrence Grade IV osteoarthritis of the right knee with severe varus deformity of 19° (Figure 1) [6].

The preoperative Knee Society Score (KSS) was 31, and the functional score was 20 [7]. After appropriate preoperative evaluation, the patient was scheduled for computed tomography (CT)-based robotic-assisted TKA (MISSO; Meril Life Sciences Pvt. Ltd., Vapi, India).

**Figure 2:**
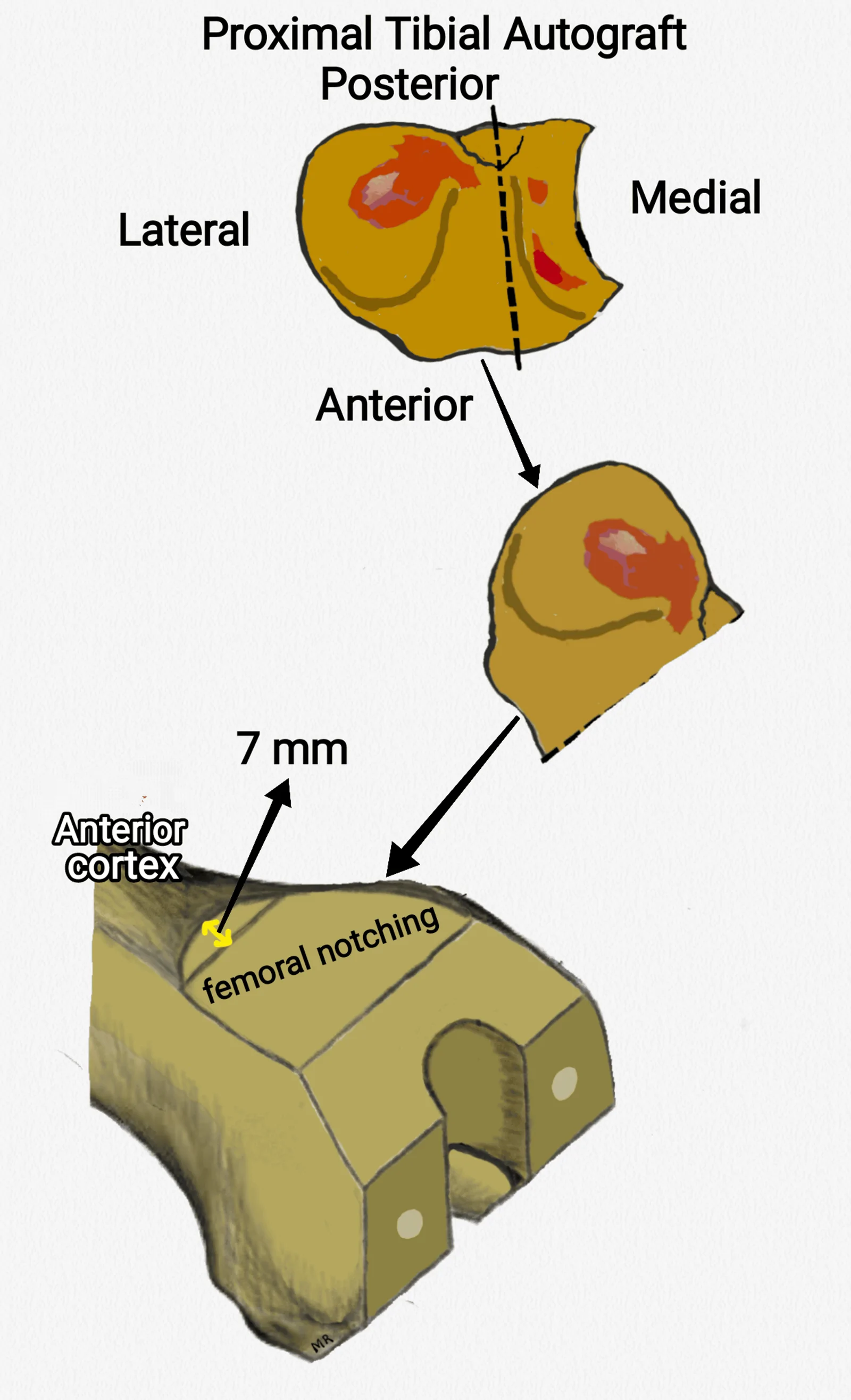
Illustration showing a proximal tibial cut autograft and femoral notching depth. The image was created by the author using the Linea Sketch app (The Iconfactory, Inc., Boston, Massachusetts, USA).

The femur was prepared first, followed by the tibia. During the very first cut of femoral preparation using the robotic arm, the anterior cortex was violated due to an unanticipated over-resection, resulting in Grade III (less than 25% of the medullary canal involvement) femoral notching with sharp edges [1]. The notching was 7 mm deep, determined using a standard sterile surgical ruler. The robotic system failed to sound an alarm or stop resecting even when bone resection exceeded the predetermined haptic boundary. The robotic system was abandoned, and the remaining femoral and tibial cuts were completed manually using conventional cutting guides. Given the compromised structural integrity of the anterior cortex, it was decided to reconstruct the defect using an autograft from the proximal tibial resection rather than using a stemmed femoral component. An appropriately sized structural graft was fashioned from the resected tibial bone (Figure 2).

**Figure 3:**
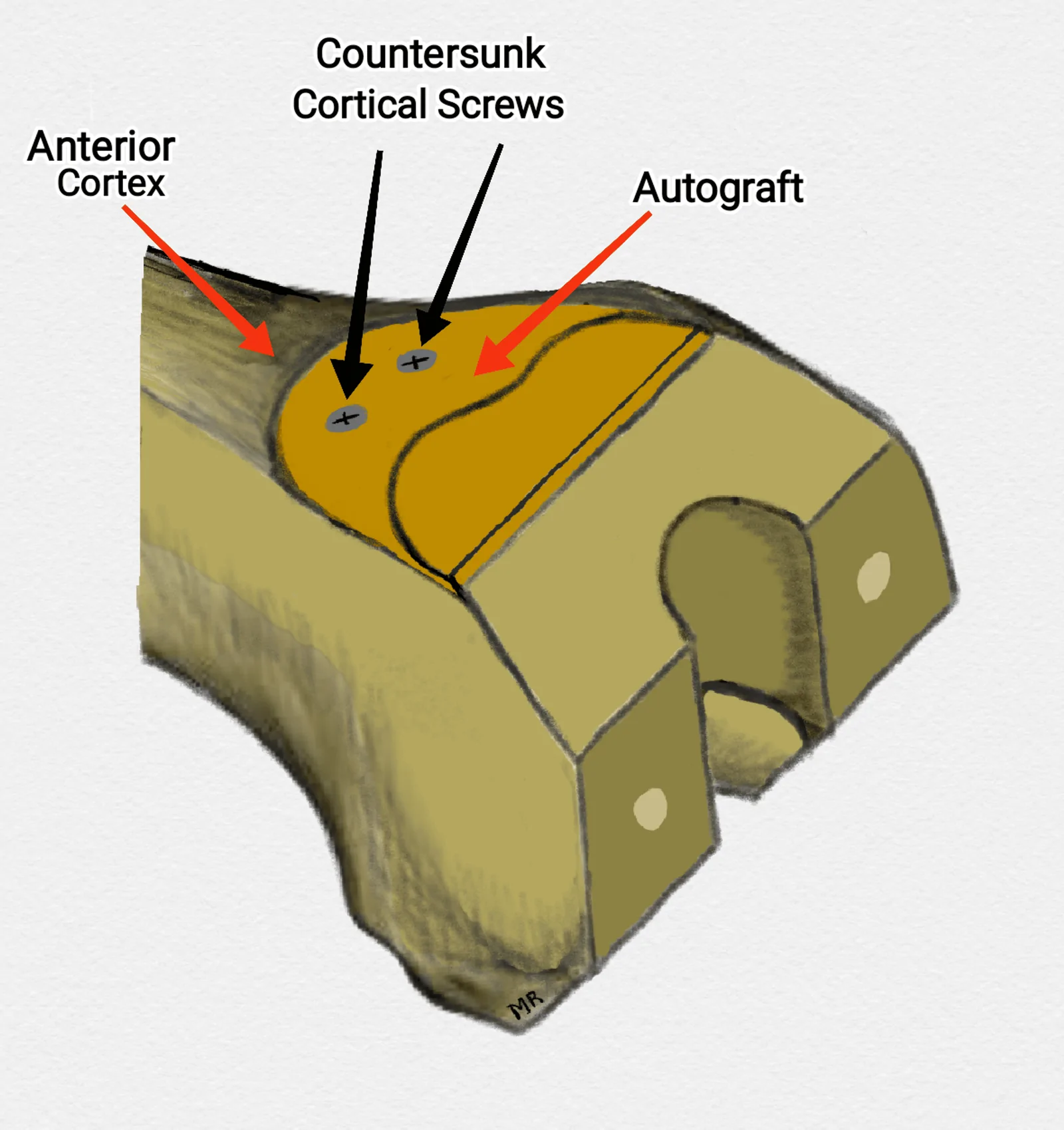
Illustration showing the tibial cut autograft fashioned to fit the femoral notching defect and fixed with two cortical screws. The image was created by the author using the Linea Sketch app (The Iconfactory, Inc., Boston, Massachusetts, USA).

The resected tibial cut bone was cleared of cartilage, trimmed, and contoured to match the dimensions of the femoral notching defect approximately. The autograft was positioned flush with the defect with the cancellous part facing the marrow, secured using two 3.5 mm cortical screws inserted in an anterior-to-posterior direction, achieving stable fixation (Figure 3).

**Figure 4:**
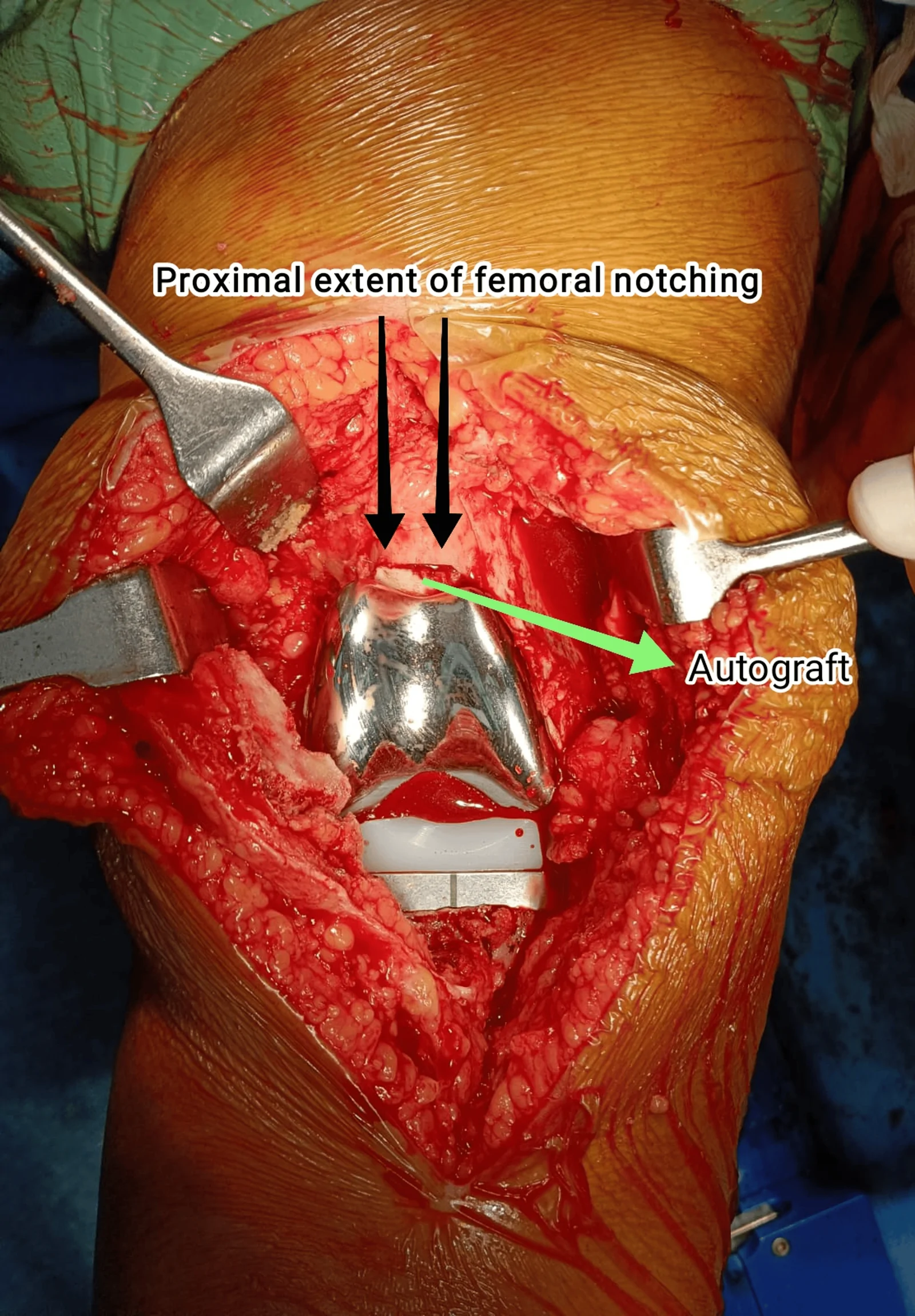
Intraoperative image after standard cruciate-retaining primary component placement showing the proximal extent of the femoral notching filled with the autograft.

Bi-cortical purchase was obtained to ensure compression, prevent bone cement from entering the graft-host bone interface, and facilitate union. The screw heads were countersunk within the graft to minimize prominence and avoid interfering with the seating of the anterior flange of the femoral component. This restored the anterior cortical contour and retained the original femoral component plan. The surface of the autograft was roughened using a rasp before cement application. The cruciate-retaining femoral and tibial components were then implanted (DESTIKNEE; Meril Life Sciences Pvt. Ltd., Vapi, India), and stability was confirmed. The edge of the femoral notching was 5 mm proximal to the proximal margin of the anterior flange of the femoral component (Figure 4).

The wound was closed in layers.

Postoperative follow-up

**Figure 5:**
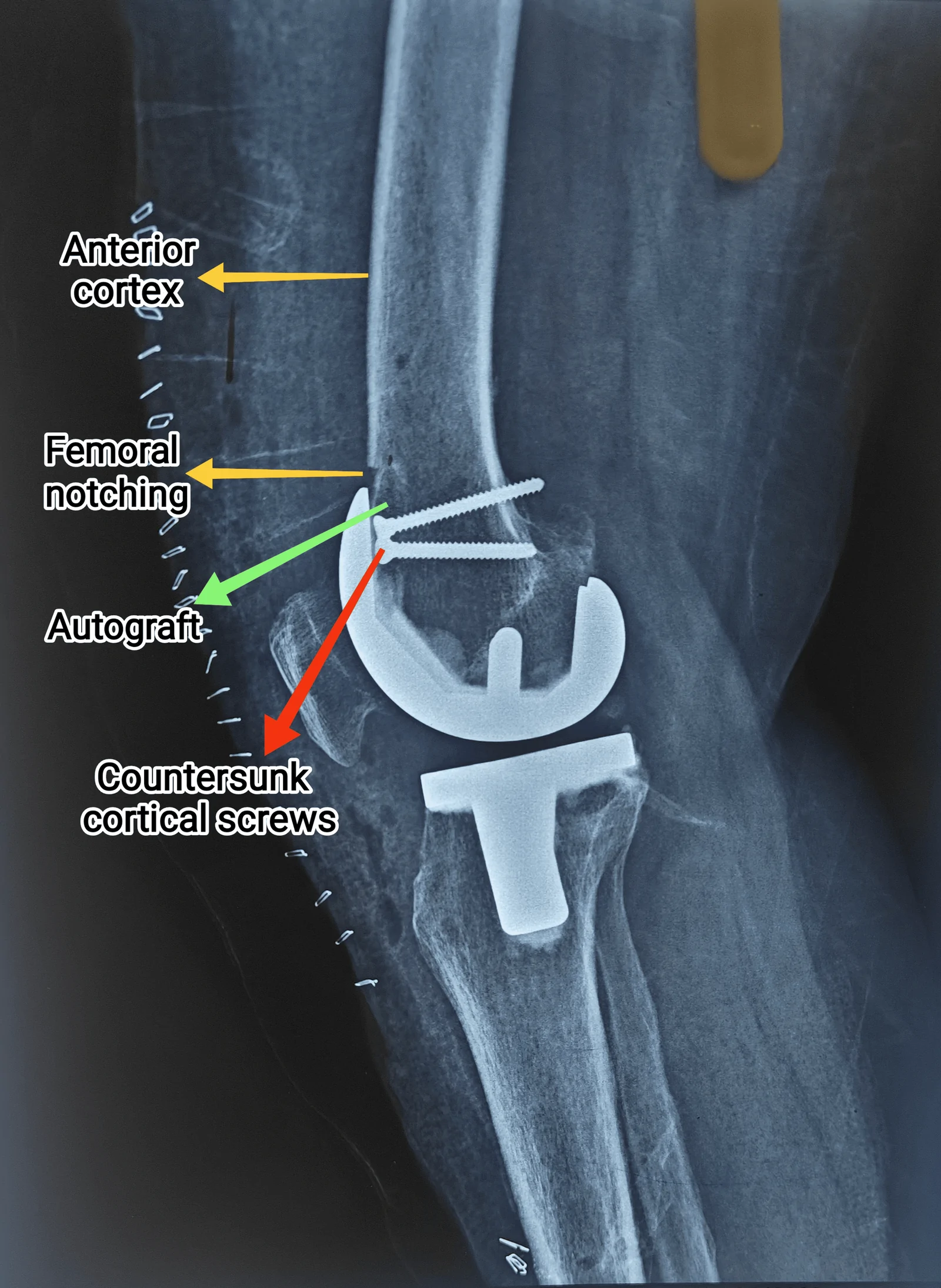
Immediate postoperative lateral-view radiograph of the right knee demonstrating Grade III femoral notching reconstructed using a structural proximal tibial cut autograft, fixed with two countersunk cortical screws, with standard cruciate-retaining primary femoral and tibial components in situ.

**Figure 6:**
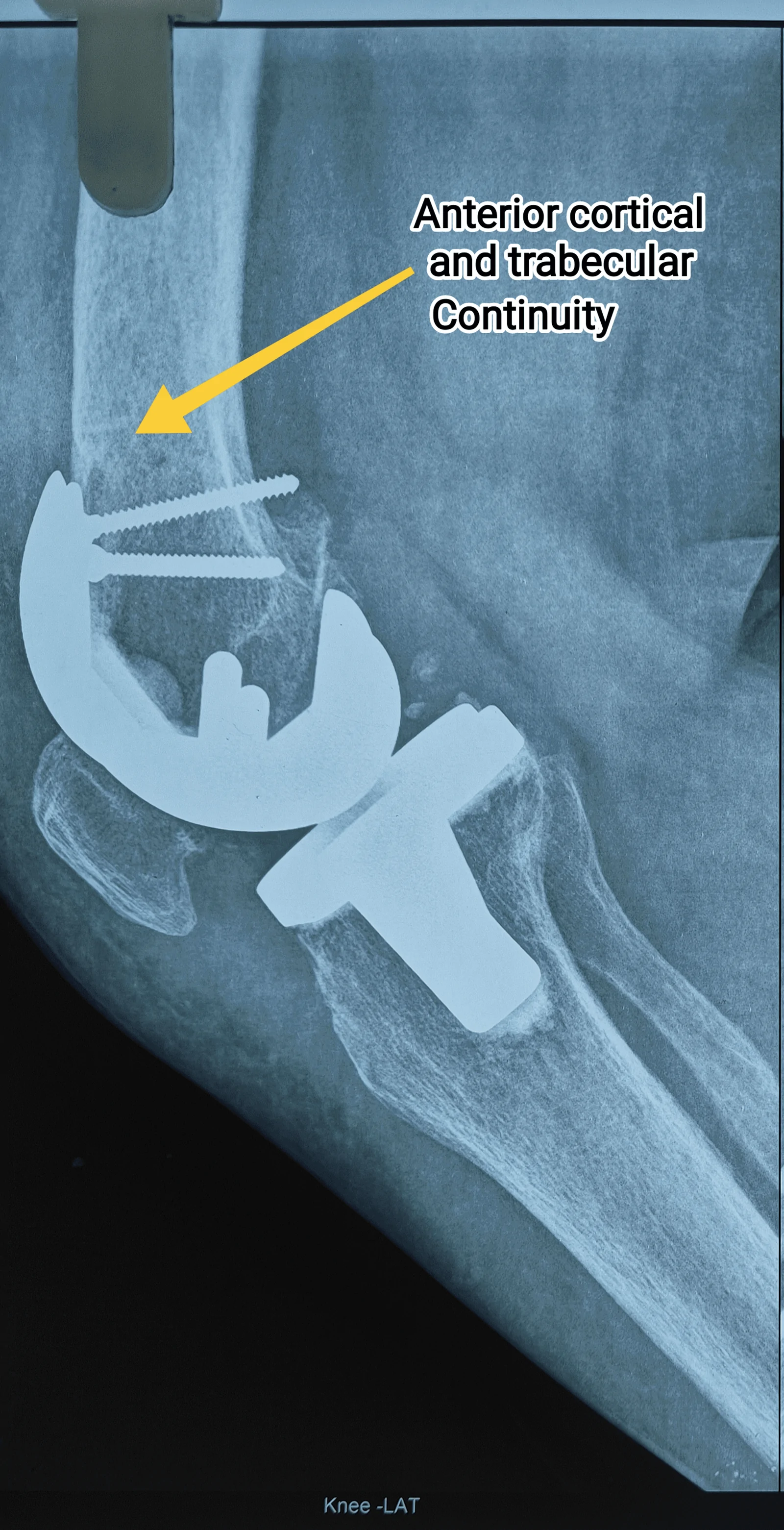
Postoperative lateral-view knee radiograph at six months showing anterior cortical and trabecular continuity, indicating autograft incorporation.

The patient was maintained on a non‑weight-bearing regimen for four weeks to avoid strain at the autograft-host interface and facilitate healing, while active knee range of motion exercises were encouraged. From the fifth week onwards, partial-weight‑bearing mobilization was permitted, progressing to full-weight-bearing mobilization from the seventh week onwards. Radiographs were obtained in the immediate postoperative period, at six weeks, 12 weeks, six months, and 12 months postoperatively (Figures 5-6).

**Figure 7:**
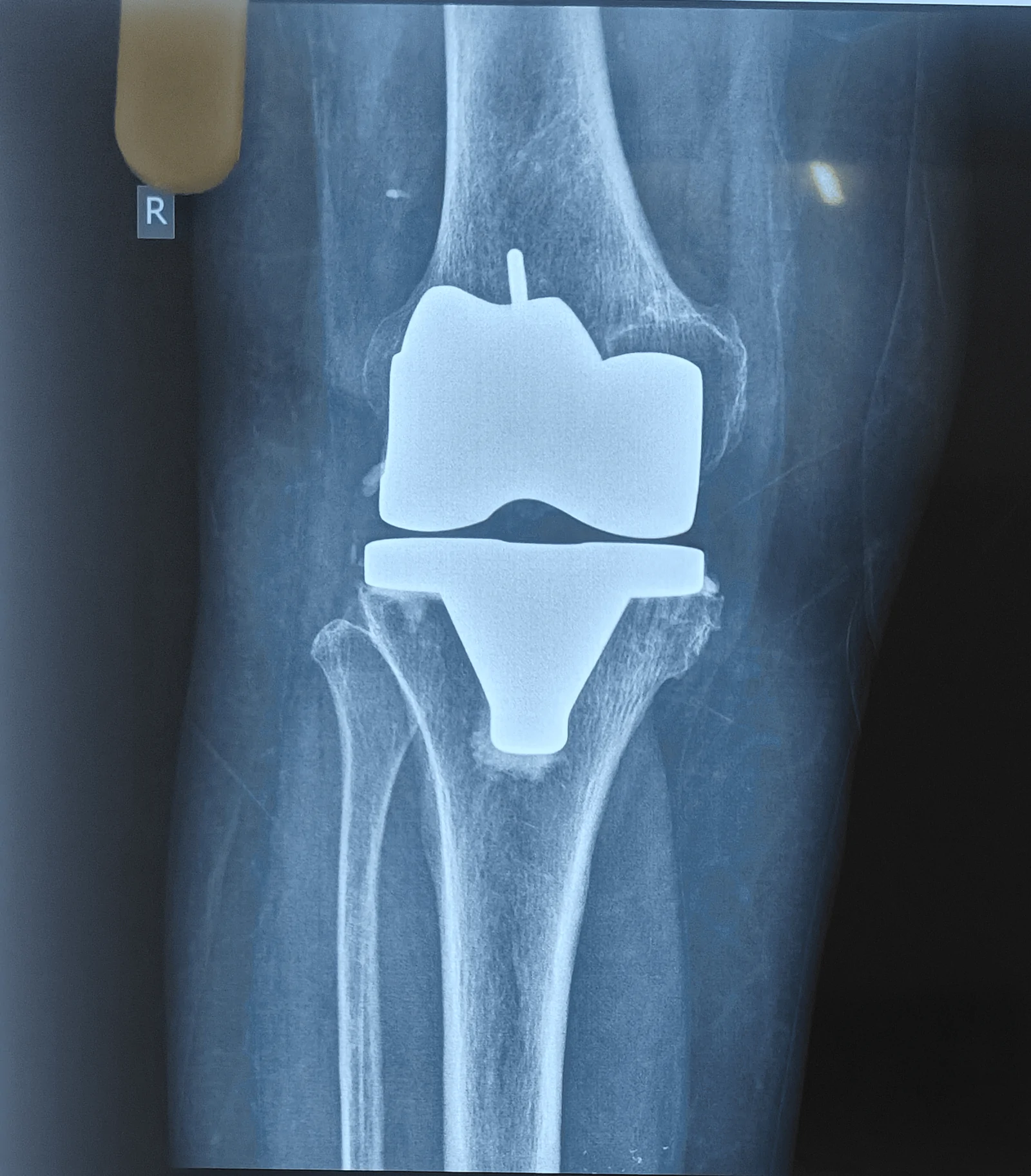
Postoperative anteroposterior-view knee radiograph at 12 months.

**Figure 8:**
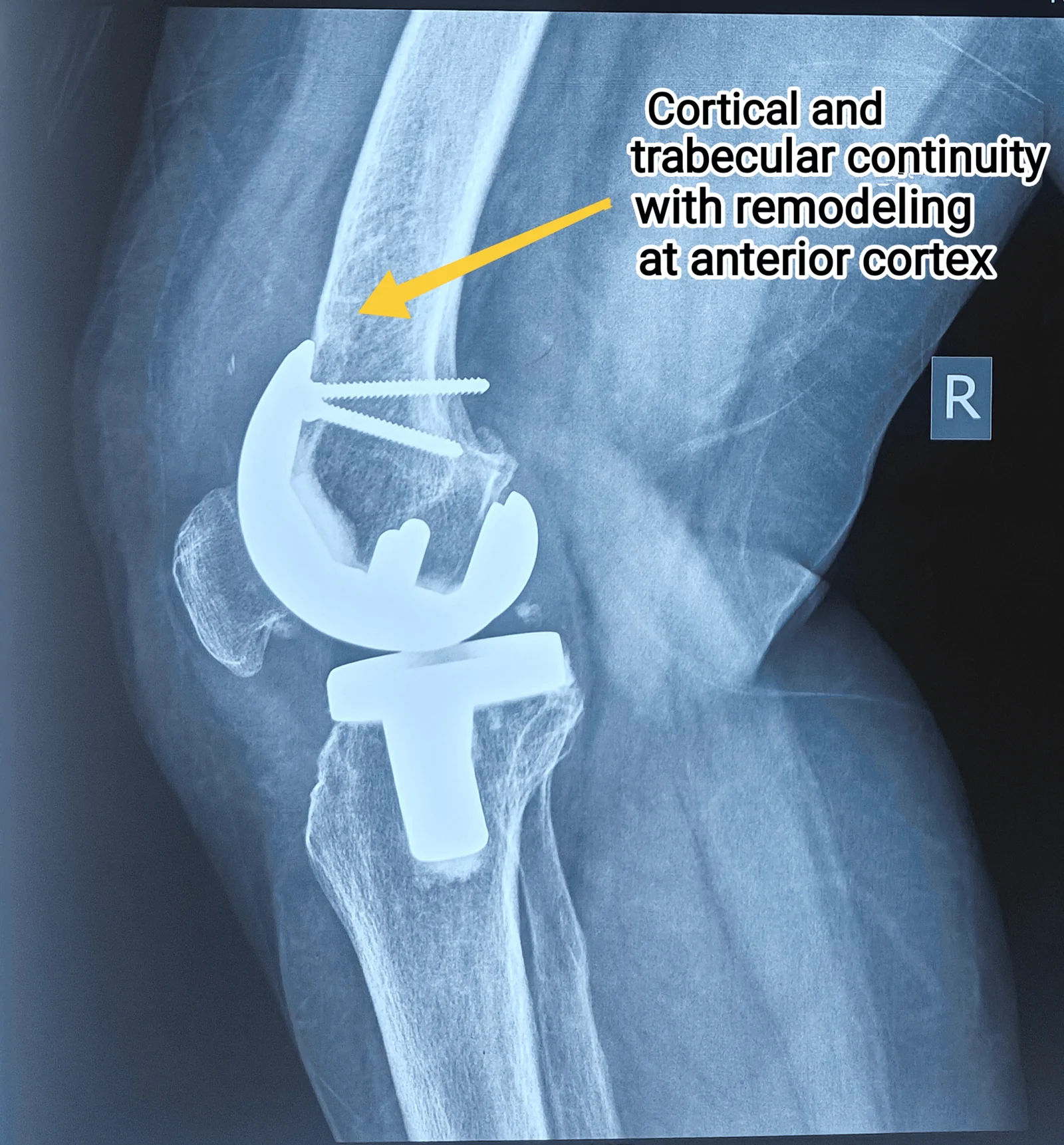
Postoperative lateral-view knee radiograph at 12 months showing anterior cortical and trabecular continuity, indicating autograft integration and remodeling.

At the 12-month follow‑up, the range of motion was 0°-0°-120°(extension-neutral-flexion). The KSS knee score was 99, and the functional score was 90. Radiographs were assessed by the treating team and showed anterior cortical and trabecular continuity, indicating autograft integration and remodeling (Figures 7-8).

## Discussion

Femoral notching is a recognized iatrogenic complication of TKA. Its incidence varies widely and is higher when navigation systems are used because of differences between anatomical and mechanical axes and the way reference cuts are planned [3,8]. One primary technical variable is that the conventional distal femoral cut is aligned with the anatomical axis, whereas navigated and robotic systems cut perpendicular to the mechanical axis [3]. Lateral and anterior bowing are associated with an increased risk of femoral notching [3]. Biomechanical work by Lesh et al. showed that a full-thickness anterior cortical defect proximal to the femoral component decreases bending strength by approximately 18% and torsional strength by nearly 40%, with fractures occurring at the notch under bending stress [9]. A meta-analysis subsequently reported that anterior notching of ≥3 mm depth is associated with a four-fold increase in the risk of periprosthetic femoral fractures after TKA [5]. Notching >3 mm with sharp corners located just proximal to the anterior flange is associated with the highest stress concentration, significantly increasing the risk of periprosthetic femoral fractures [2].

In the present case, the defect was classified as Tayside Grade III, indicating extension up to 25% of the medullary canal [1]. Given the depth of the defect and disruption of the anterior cortex, we elected to provide structural reconstruction rather than leave the notch unsupported. Various strategies have been proposed to address significant notching. The use of a stemmed femoral component bypasses the weakened metaphysis and facilitates stress distribution, but increases implant costs and the risk of fat embolism [2,10]. Biomechanical studies have favored the use of a femoral stem, as strain levels at the notch edge were found to be higher than those of an intact femur [11]. Some authors have suggested extending the anterior cut proximally such that the notch edge is >15 mm proximal to the anterior flange and rounding any sharp corners with a burr or saw [2]. Some authors have accepted moderate notching without structural reinforcement, reporting no or low fracture rates at medium-term follow-up, but most of these series included less severe defects than in the present case [1,8,12].

The proximal tibial autograft was chosen instead of a stemmed femoral component because of cost constraints and the additional steps required for stem insertion. Further proximal resection and rounding the margins would remove additional bone and further weaken the cortex. Given the grade III femoral notching, protected weight bearing would restrict mobility and prolong rehabilitation. The technique described in this report offers several advantages. The autograft is immediately available without any additional procedure, avoiding the use of an allograft, stem, or augments, and it successfully reconstructs anterior cortical continuity. The use of standard primary TKA components avoided the complexity of converting to a stemmed implant. At 12 months, radiographically, the graft appeared well integrated, and the patient was clinically doing well.

However, this report describes only a single patient with a 12-month follow‑up. No advanced imaging, such as a CT scan, was used to accurately confirm graft union, and no biomechanical validation of the construct was performed. The true effect of this reconstruction on subsequent fracture risk and revision surgery is unknown. Whether utilizing a structural bone graft from other donor sites or an allograft provides a similar outcome remains to be evaluated in future studies. Despite these limitations, this case highlights a simple, cost‑effective option to manage significant anterior femoral notching. Nevertheless, the decision to utilize a proximal tibial autograft should be individualized, and larger case series with longer follow‑up are required before recommendations can be made.

## Conclusions

Femoral notching during primary TKA may compromise the mechanical integrity of the distal femur and increase the risk of a future supracondylar periprosthetic fracture. In this patient, a Grade III femoral notch was reconstructed using a structural autograft from the proximal tibial resection, secured with countersunk cortical screws, followed by the implantation of standard cruciate-retaining primary components, with favorable clinical and radiographic findings at 12 months. This case demonstrates the technical feasibility of this reconstruction approach; however, its safety, durability, and effect on long-term fracture risk require evaluation in larger cohorts with longer follow-up.
